# Visual outcomes of sight threatening radiation-induced meningiomas after low-dose head irradiation: tinea capitis as a paradigm

**DOI:** 10.1007/s11060-026-05696-z

**Published:** 2026-07-07

**Authors:** Dahlia Palevski, Hadas Stiebel-Kalish, Omer Yizhach Bialer

**Affiliations:** 1 Ophthalmology Department, Rabin Medical Center, 39 Jabotinski St., Petach-Tikva, Israel; 2 Gray Faculty of Medical and Health Sciences, Tel-Aviv University, Tel-Aviv, Israel; 3 Laboratory of Eye Research, Felsenstein Medical Research Center, Tel Aviv University, Petah-Tikva, Israel

**Keywords:** Radiation, Meningioma, Tinea capitis, Radiation-induced meningioma

## Abstract

**Purpose:**

To investigate the visual characteristics and prognosis of vision-threatening meningiomas following low dose head radiation and compare them to those of primary unexposed meningiomas.

**Methods:**

A retrospective cohort of adults with meningiomas adjacent to the optic nerves or intracranial visual pathways was categorized into radiation-exposed and unexposed meningiomas. Data collected included demographics, visual biomarkers at presentation and at final follow-up, including Visual Acuity (VA), color vision (CV), Visual Field (VF) and peripapillary Retinal Nerve Fiber Layer (RNFL) thickness via Optical coherence tomography (OCT). Comparative analyses were performed between radiation-exposed and unexposed cohorts.

**Results:**

289 eligible patients seen between 2004 and 2023 were included, out of which 31 (10.7%) had undergone tinea capitis radiation. At presentation, patients exhibited a median BCVA of 0.1 LogMAR [IQR 0.1–0.6], median CV of 100% [IQR 64.5%-100%], and 9 (29%) had normal visual fields. Notably, 16 (51.61%) experienced visual loss. Their BCVA decreased at final follow-up, to median 0.23 [IQR 0.1–1.46]. Comparative analysis with 114 radiation-naïve patients with meningioma-induced visual loss revealed no statistically significant disparities in visual characteristics at presentation or at follow-up.

**Conclusions:**

No statistically significant differences in clinical presentation or visual prognosis were detected between patients with visual pathway meningiomas exposed to low-dose childhood radiation and radiation-naïve patients. Since low-dose cranial irradiation continues to be widely used for conditions such as thyroid eye disease, our findings are relevant beyond the specific context of tinea capitis.

**Supplementary Information:**

The online version contains supplementary material available at 10.1007/s11060-026-05696-z.

## Introduction

Radiotherapy has been used for over a century [1]. It is broadly classified as low-dose (< 10 Gy) and high-dose treatment. Low-dose irradiation has historically been applied in several benign and inflammatory disorders, including thyroid-associated orbitopathy (TAO) [2], orbital pseudotumor [3], cutaneous T-cell lymphoma (mycosis fungoides) [4], primary cutaneous B-cell lymphoma [5], postoperative keloid control [6], neovascular age-related macular degeneration [7], and even Alzheimer’s disease [8]. 

Another historical application was the treatment of tinea capitis (scalp ringworm). Before the introduction of griseofulvin in 1960, scalp irradiation was the treatment of choice worldwide [9]. Children across the United States, United Kingdom, Portugal, Poland, Ukraine, the former Yugoslavia, Australia, North Africa, and the Middle East routinely underwent this procedure. It is estimated that more than 200,000 individuals were exposed between the 1940s and 1950s [9, 10]. Although the practice was later abandoned due to recognized long-term risks, these cohorts provide critical insight into the late effects of childhood irradiation.

Extensive follow-up has demonstrated that low-dose cranial irradiation increases the risk of both malignant and benign tumors [11]. The relative risk of meningiomas, in particular, is nearly tenfold higher among irradiated individuals compared with controls [11]. Meningiomas are the most common primary intracranial tumor in adults, representing up to 41% of cases [12]. Established risk factors include obesity, cigarette smoking, estrogen therapy, hypertension, and ionizing radiation, with childhood cranial irradiation emerging as an association [12, 13]. 

Radiation-induced meningiomas differ from sporadic meningiomas in several respects: they occur at a younger age, are more likely to arise from the calvaria, frequently present as multiple lesions (meningiomatosis), and have been reported to carry a higher risk of recurrence [13, 14]. Despite these distinctive features, little is known about their impact on visual function. Meningiomas along the visual pathways or occipital cortex, although rarely life-threatening, can cause irreversible visual loss and significant disability [15]. 

To date, no comparative study has systematically examined whether vision threatening meningiomas associated with prior low-dose irradiation behave differently from those in radiation-naïve patients. The present study addresses this gap by evaluating the demographic, neuro-surgical, and neuro-ophthalmological features of patients with vision threatening meningiomas. Our cohort focuses on individuals irradiated for tinea capitis, but the findings are applicable to other populations exposed to low-dose cranial or orbital radiotherapy. Understanding the visual consequences of radiation-related meningiomas remains highly relevant today, as low-dose cranial irradiation continues to be used worldwide for several benign conditions.

## Methods

This retrospective case-control study was approved by the local Institutional Review Board. The requirement for informed consent was waived due to the retrospective nature of the study.

### Study cohort

The medical records of all patients referred to the Neuro-Ophthalmology Unit of a tertiary medical center between January 2004 and March 2023 were reviewed to identify patients with “sight-threatening meningiomas”, defined as tumors anatomically adjacent to the optic nerve, optic chiasm, optic radiations, or primary visual cortex. The primary study group included those patients who reported previous treatment with low-dose radiation for tinea capitis (the “radiation group”) while patients without prior radiation exposure formed the “control group”.

### Exclusion criteria

Patients with a history of brain radiation for the treatment of head and neck tumors or systemic malignancies, or with visual loss attributable to etiologies unrelated to the meningioma (e.g., advanced glaucoma) or with insufficient data regarding meningioma characteristics (e.g., anatomic location, treatment modality) were excluded. Only patients with a full neuro-ophthalmological examination were included.

### Data collection

We collected the following data on tumor characteristics: (1) date of diagnosis (defined as the first neuroimaging showing the meningioma; if unavailable, the year of first documentation in medical records), (2) the number and location of meningiomas, (3) Maximal tumor diameter (defined as the largest reported tumor dimension in the neuroimaging report) and (4) treatment modality: surgery (craniotomy or trans-sphenoidal excision), fractionated stereotactic radiosurgery (FSR), single-dose stereotactic radiosurgery (SRS) or a combination of them. Tumor-related imaging characteristics were extracted from radiology reports and clinical records; neuroimaging studies were not re-reviewed centrally for the purposes of this study.

The following visual parameters were recorded for the initial and final neuro-ophthalmological examinations:

*Best Corrected Visual Acuity* (BCVA) was measured using Snellen chart at 20 feet, with usual refractive correction and pinhole when necessary. BCVA was converted to LogMAR for statistical analysis. Non-numeric visual acuities were assigned the following LogMAR in accordance with previous publications: [16] No Light Perception (NLP) = 3.0, Light Perception (LP) = 2.3, Hand Motion (HM) = 2.0, and Finger Counting (FC) = 1.7. For visual acuity tested at 3 feet: 3/18 − 3/36 = 1.40 LogMAR, 3/54 − 3/72 = 1.54 LogMAR, 3/108-3/180 = 1.65 LogMAR.

*Color Vision* (CV) was assessed using Ishihara or Hardy-Rand-Rittler (HRR) pseudoisochromatic plates. The number of correct plates was converted into percent plates correct (e.g., 10/12 Ishihara plates correct in the right eye = 83.3% OD).

Optic disc appearance was documented via dilated fundoscopy as normal, pale, or edematous.

*Humphrey Visual Fields* (HVF): Tests were performed using the Humphrey Visual Field Analyzer III (Carl Zeiss Meditec, Irvine, CA, USA). SITA Fast or SITA Standard strategies were employed for most patients, while the FASTPAC strategy with stimulus V was used for those with very low vision. Only HVF tests conducted within a 3-month window before or after the ophthalmological examination were included in the analysis. Due to incomplete documentation of Mean Deviation (MD) across different strategies, visual field defects were recorded as free-text descriptions.

*Optical Coherence Tomography* (OCT): Average peripapillary Retinal Nerve Fiber Layer (pRNFL) thickness measurements were obtained using either the Cirrus Spectral Domain (SD)-OCT (Zeiss Meditec, Irvine, CA, USA) or the Spectralis SD-OCT (Heidelberg Engineering GmbH, Heidelberg, Germany). Only OCT scans performed within a 3-month window before or after the ophthalmological examination and with good reliability were included in the analysis. To allow for comparison of pRNFL measurements between the two devices, we applied the following formula as recommended by Kenney et al.: [17]

$$\eqalign{ & {\rm{Cirrus - average - thickness}} \cr & {\rm{ = - 5}}{\rm{.05}}\>{\rm{m + Spectralis - average - thickness}} \cr}$$

Ganglion Cell Layer (GCL) thickness measurements were extracted when available; however, because GCL acquisition was not routinely available throughout the study period and was not consistently obtained across OCT platforms, these data were too sparse for meaningful statistical analysis and were therefore not included in the primary analyses.

Final examination data were included only for patients who completed at least 6 months of follow-up.

Because no universally accepted threshold-based definitions for optic neuropathy in general, or compressive optic neuropathy in particular, exist in the literature [18–21], operational definitions were developed for this retrospective study to provide a standardized framework for classifying visual pathway involvement. Based on initial visual characteristics, patients were categorized as either having or not having visual damage, according to the following criteria:

1. No visual loss: No evident visual impairment or structural damage.
2. Clinical Visual Loss (CVL): Visual loss corresponding to the side affected by the meningioma, defined by two or more of the following:

- BCVA ≥ 0.17 LogMAR (approximately Snellen 20/30).
- CV ≤ 83.3% (approximately 10/12 Ishihara plates correct).
- Relative afferent pupillary defect (RAPD).
- Optic disc pallor or edema.
- Visual field defect (excluding non-specific or small peripheral scotomas, which were considered normal).

3. Subclinical Anterior Visual Pathway Damage (SAPD): Absence of visual symptoms, with visual acuity and color vision better than the thresholds defined for CVL and normal visual field testing, but with objective evidence of anterior visual pathway injury manifested by pRNFL or macular Ganglion Cell Layer (mGCL) thinning on OCT corresponding to the side affected by the meningioma with or without ipsilesional optic disc pallor

Conservative visual acuity and color vision thresholds were selected to improve specificity and reduce false-positive classification.

### Statistical analysis

Continuous variables were summarized using median (IQR) due to small sample size and non-normality, except for age and RNFL thickness, which were normally distributed and reported as mean ± SD. Categorical variables were presented as frequencies and percentages. For bilateral cases, only the worse eye was analyzed to permit patient-level rather than eye-level comparisons, ensuring that visual outcomes were represented by a single value per patient and avoiding treatment of two eyes from the same patient as statistically independent observations. In addition, selecting the worse eye allowed comparison of the maximum visual deficit experienced by patients in each group. Optic disc and HVF findings were dichotomized as normal or abnormal. Between- and within-group comparisons used Wilcoxon signed-rank tests for continuous data and chi-square, Fisher’s exact, or McNemar’s tests for categorical data, with Holm–Bonferroni correction applied. Analyses were performed in R (R Foundation for Statistical Computing, Vienna, Austria, https://www.R-project.org).

## Results

Our review of medical records identified 386 patients with a diagnosis of meningioma. Supplemental Fig. 1 presents a study flow diagram illustrating the selection and exclusion process for the study cohort. Overall, 325 adult patients with vision threatening meningiomas were evaluated at the Neuro-Ophthalmology Unit between January 2004 and March 2023. Of these, 37 (11.6%) reported childhood exposure to low-dose radiation for the treatment of tinea capitis, confirmed by the national registry. 282 patients had no history of radiation exposure. Following the exclusion process, the final analysis included two groups: the radiation group, consisting of 31 patients previously treated with low-dose scalp radiation for tinea capitis, and the control group, consisting of 258 patients without prior radiation exposure.

### Patient characteristics

Table 1 summarizes the demographics of both groups. No significant differences were observed in gender distribution, age at diagnosis, or duration of follow-up. However, patients in the radiation group were slightly older at their first visit.

Table 2 presents the neurosurgical characteristics of the tumors in both groups. Meningiomatosis—defined as multiple meningiomas in a single patient—was significantly more common in the radiation group (Odds Ratio [OR] = 4.6; 95% CI: 2.07–10.2; χ² (1, *N* = 289) = 14.09, *p* = 0.0001). There were no significant differences in the anatomical distribution of tumors, maximal tumor diameter, or in the treatment modalities applied.

The interval between meningioma diagnosis and the first neuro-ophthalmological evaluation ranged from three months prior to diagnosis to 31.5 years afterward (median: 32 months; see Table 1). A detailed comparison of visual characteristics in the radiation group between first and last follow-up exam is provided in Supplemental Table 1.

**Table 1:** Demographics and neuro-ophthalmological follow-up data

	Radiation	Controls	*p* value
**Number of patients (%)**	31 (10.7%)	258 (89.3%)	
**Sex**			
Women, n (%)	27 (87.1%)	210 (81.4%)	0.594
**Age** (years)			
At initial N-O examination Mean ± SD	66.22 ± 7.3	60.18 ± 15.0	0.028
At diagnosis of meningioma Mean ± SD	59.7 ± 11	57.9 ± 14.8	0.524
**Time from diagnosis to 1st neuro-ophthalmic exam** (months)			
Median [IQR]	32 [6.5–102]	6.5 [2–27]	0.001
**Follow-up** (months)			
Median [IQR]	77 [ 21.7–142]	51.6 [24.8–121]	0.372
**Diagnosis of meningioma made after neuro-ophthalmological exam**			
Yes, n (%)	1 (3.2%)	15 (5.8%)	0.857

Demographics and neuro-ophthalmological follow-up data. IQR= interquartile range

N−O = neuroophthalmological. SD= standard deviation

Although the interval between meningioma diagnosis and the first neuro-ophthalmological evaluation was significantly longer in the radiation group, additional sensitivity analyses demonstrated no significant association between this interval and the presence of visual loss or baseline visual acuity at the initial neuro-ophthalmological examination.

**Table 2:** Neuro-surgical characteristics of radiation and control groups

	Radiation	Controls	*p* value
**Number of patients (%)**	31 (10.7%)	258 (89.3%)	Na
**Number of meningiomas per patient**, mean ± SD	1.7 ± 1	1.25 ± 0.8	0.004
**Maximal tumor diameter (mm)**, median [IQR]	24 [15.5–33.5]	23 [13–36]	0.694
**Anatomic location of the meningioma** ^a^			
Anterior clinoid process	10 (32.3%)	72 (27.9%)	0.364
Cavernous sinus	3 (9.7%)	37 (14.7%)	
Sphenoid wing	6 (19.4%)	29 (11.2%)	
Tuberculum sella / Sella Turcica	3 (9.7%)	38 (14.7%)	
Planum sphenoidale / Olfactory groove	2 (6.5%)	26 (10.1%)	
Parietal / temporal / occipital lobe	3 (9.7%)	16 (6.2%)	
ONSM	0	8 (3.1%)	
Orbital	1 (3.2%)	7 (2.7%)	
Multiple locations	3 (9.7%)	8 (3.1%)	
Other ^b^	0	18 (7%)	
**Treatment**			
No treatment	11 (35.5%)	96 (37.2%)	0.162
Craniotomy	9 (29%)^c^	85 (32.9%) ^d^	
Radiation	3 (9.7%)	46 (17.8%)	
Craniotomy + radiation	8 (25.8%)^e^	31 (12%)^f^	
**Segment of the visual apparatus adjacent to the meningioma**			
Optic nerve	23 (74.2%)	193 (74.8%)	0.03
Chiasm	3 (9.7%)	44 (17%)	
Optic radiations	2 (6.4%)	14 (5.4%)	
Occipital cortex	1 (3.2%)	6 (2.3%)	
Multiple sites	2 (6.4%)	1 (0.4%)	

Radiation treatment includes both fractionated stereotactic radiation and stereotactic radiosurgery. a refers to the vision threatening meningioma when multiple meningiomas were present. b other locations include Petro clival, posterior fossa, frontal lobe, and Meckel’s cave, each with no more than six patients overall. c One patient underwent multiple craniotomies. d Nine patients underwent multiple craniotomies; seventy−seven patients had a single craniotomy. e Two patients underwent multiple craniotomies; six patients had a single craniotomy. f Twenty−nine patients underwent a single craniotomy and radiation; one patient had transsphenoidal surgery and radiation. IQR = Interquartile Range, ONSM = Optic Nerve Sheath Meningioma. SD= standard deviation

### Comparison of visual findings between radiation and control groups

The intergroup comparison included only patients with visual loss due to meningioma compression. Of the 16 affected radiation patients, 14 (87.5%) completed at least six months of follow-up. Among controls, 102 of the 114 affected patients (89.5%) had similar follow-up.

Table 3 compares visual characteristics at the first examination, and Table 4 compares them at final follow-up. Across both time points, patients exposed to radiation showed similar BCVA, CV, optic disc appearance, HVF findings, and pRNFL thickness compared to controls.

**Table 3:** Comparison of visual characteristics in the affected eye at the initial neuro-ophthalmological examination

	Radiation	Controls	*p* value
*N*	16	114	
**Classification of visual loss in the worse eye**			
Clinical visual loss, n (%)	14 (87.5%)	103 (90.3%)	0.732
Subclinical Anterior Visual Pathway Damage, n (%)	2 (12.5%)	11 (9.7%)	
**Best corrected visual acuity**, ***LogMAR***			
Missing	0	0	
Median [IQR]	0.1 [0.1–0.6]	0.15 [0.04–0.5]	0.867
**Color vision**,** % plates correct**			
Missing	0	3 (2.6%)	
Median [IQR]	65% [0%-100%]	100% [0%-100%]	0.679
**RAPD**			
Missing	0	1 (0.9%)	
n (%)	11 (68.8%)	71 (62.8%)	0.855
**Optic disc appearance**			
Missing	0	6 (5.3%)	
Normal, n (%)	6 (37.5%)	34 (29.8%)	0.465
Pallor, n (%)	10 (62.5%)	71 (62.3%)	
Edema, n (%)	0	9 (7.9%)	
**Humphrey visual fields**			
Missing, n (%)	2 (11.8%)	23 (20.2%)	
Normal, n (%)	3 (21.4)	17 (17.5%)	0.922
Nonspecific VF defect, n (%)	1 (7.1%)	3 (3.1%)	
Abnormal, n (%)	10 (71.4%)	76 (78.4%)	
**Average peripapillary RNFL thickness**,** µm**			
Missing, n (%)	13 (81.2%)	84 (73.7%)	
Mean ± SD	77.9 ± 6.3	75.2 ± 13.1	0.554

Visual characteristics in the radiation and control groups at their initial neuro−ophthalmological evaluation is presented. When both eyes were affected by the meningioma, the worst−seeing eye was chosen for analysis. IQR= interquartile range. SD= standard deviation. VF = visual field. RAPD = Relative Afferent Pupillary Defect. RNFL= Retinal Nerve Fiber Layer

**Table 4:** Comparison of visual characteristics in the affected eye at the last follow-up

	Radiation	Controls	*p* value
*N*	14	102	
**Best corrected visual acuity**, ***LogMAR***			
Missing	0	3 (2.9%)	
Median [IQR]	0.23 [0.1–1.46]	0.18 [0.04–0.43]	0.380
**Color vision**,** % plates correct**			
Missing	0	12 (11.8%)	
Median [IQR]	100% [69%-100%]	100% [8.3%-100%]	0.562
**RAPD**			
Missing	1 (7.1%)	6 (5.9%)	
n (%)	10 (76.9%)	73 (76%)	1.0
**Optic disc appearance**			
Missing	0	6 (5.9%)	
Normal, n (%)	4 (28.6%)	20 (20.8%)	0.712
Pallor, n (%)	10 (71.4%)	74 (77.1%)	
Edema, n (%)	0	2 (2.1%)	
**Humphrey visual fields**			
Missing, n (%)	1 (7.1%)	20 (19.6%)	
Normal, n (%)	4 (30.8%)	16 (19.3%)	0.774
Nonspecific VF defect, n (%)	0	1 (1.2%)	
Abnormal, n (%)	9 (69.2%)	66 (79.5%)	
**Peripapillary RNFL thickness (µm)**			
Missing	6 (42.8%)	31 (30.4%)	
Mean ± SD	65.5 ± 14.4	65 ± 17.9	0.947

Visual outcomes at the last neuro−ophthalmological examination for patients with visual loss secondary to meningioma compression who completed at least six months of follow−up. For patients with bilateral meningioma involvement, data from the worst−seeing eye was used for analysis. IQR= interquartile range. SD= standard deviation. VF = visual field. RAPD = Relative Afferent Pupillary Defect. RNFL= Retinal Nerve Fiber Layer

### Comparison of visual characteristics between initial and final neuro-ophthalmological examinations

**Fig. 1:**
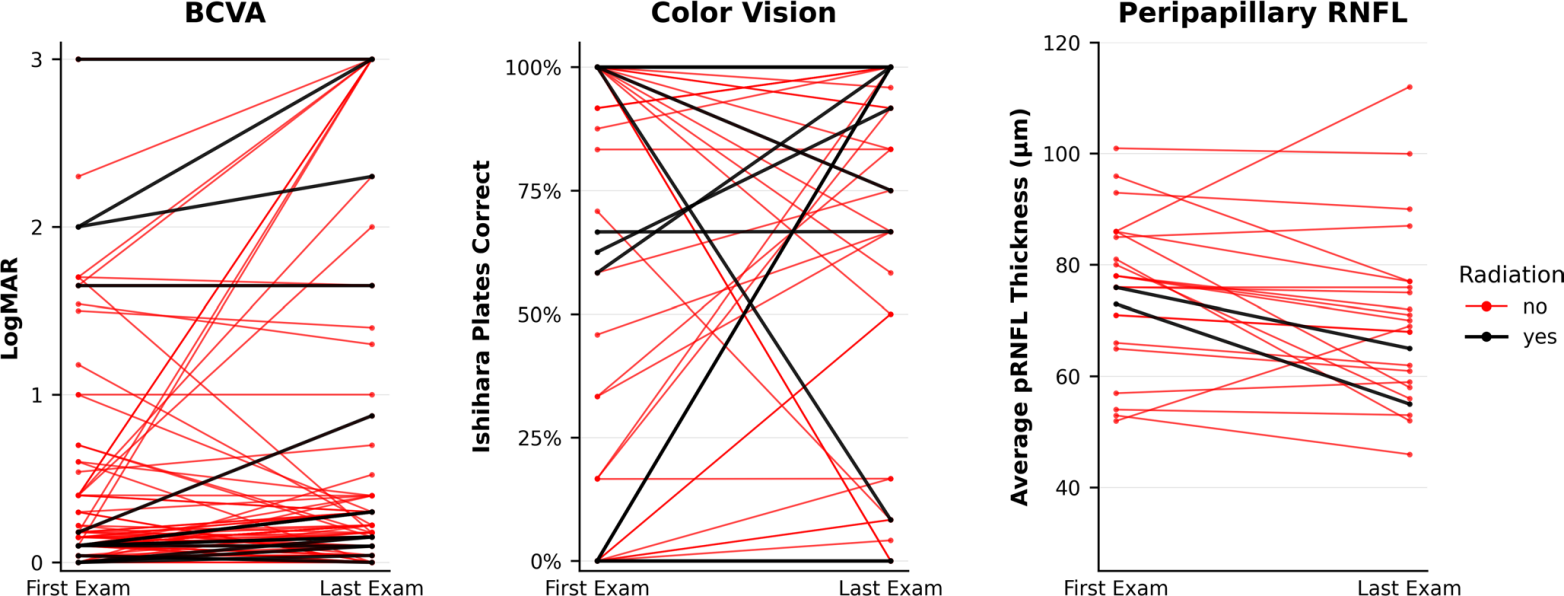
Longitudinal Changes in BCVA, CV and RNFL Between Initial and Final examinations This figure illustrates changes in visual acuity, color vision, and average peripapillary retinal nerve fiber layer thickness (measured by spectral-domain OCT) between the initial and final neuro-ophthalmological examinations. Patients previously exposed to radiation for tinea capitis are shown with thick black lines, while radiation-naïve are represented by thin red lines. BCVA panel (left), Color Vision (center), pRNFL Thickness (right). BCVA = Best-Corrected Visual Acuity; pRNFL = peripapillary Retinal Nerve Fiber Layer

We compared changes in visual parameters between the initial and final evaluations within each group, restricted to patients with visual loss due to meningioma. Figure 1 illustrates the changes in BCVA, CV, and pRNFL thickness.

In the radiation group, BCVA declined from a median of 0.1 LogMAR [IQR: 0.09–0.58] to 0.23 LogMAR [IQR: 0.11–1.46] (*p* = 0.00532; Holm-adjusted *p* = 0.026). In contrast, BCVA remained stable in the control group (median 0.15 [IQR: 0.04–0.5] at baseline vs. 0.18 [IQR: 0.04–0.43] at follow-up; *p* = 0.6443). In both groups, CV remained unchanged (100% plates correct; *p* = 0.271 for the radiation group and *p* = 0.1986 for controls), and the proportion of patients with normal VFs did not differ significantly (*p* = 1.0 for both groups).

There were no significant changes in the prevalence of RAPD (*p* = 0.6171) or optic atrophy (*p* = 0.6831) between the initial and final visits in the radiation group. However, in the control group, optic atrophy increased from 62.3% to 77.1% (*p* = 0.00104; Holm-adjusted *p* = 0.005).

The mean pRNFL thickness was lower at the final examination in both groups. However, longitudinal OCT data were limited, particularly in the radiation group, where only two patients had paired RNFL measurements available.

## Discussion

In this cohort of 325 patients with vision threatening meningiomas, we did not detect evidence that low-dose radiation exposure was associated with a more aggressive course or a worse visual outcome compared to radiation-naïve cases.

Prior literature suggests that radiation-induced meningiomatosis follows a more aggressive course, with a greater number of tumors, faster growth, and a higher proportion of atypical or anaplastic meningiomas [14, 22]. In line with these findings, we hypothesized that prior low-dose scalp irradiation would result in worse visual prognosis in patients with meningiomas involving the visual pathways. A secondary aim was to describe the neuro-ophthalmological characteristics of these patients, as this has not been previously reported.

Contrary to our hypothesis, visual outcomes did not differ significantly between groups at initial or final evaluations. BCVA, CV, RAPD, optic disc pallor, and HVF severity were all comparable. In the radiation group, median visual acuity worsened slightly over a median follow-up of 6.4 years (from 0.1 LogMAR to 0.23 LogMAR), whereas vision remained stable in the control group. This may suggest a trend toward more aggressive vision loss in radiation-induced meningiomas. However, this trend was not supported by other visual parameters, which did not exhibit consistent decline over time.

Although visual loss was numerically more common in the radiation group than in controls (51.6% versus 44.2%), this difference was not statistically significant. Therefore, our data do not support the conclusion that prior low-dose radiation exposure was associated with a greater likelihood of visual impairment at presentation. Nevertheless, given the limited sample size of the radiation cohort, we cannot exclude the possibility of a modest increase in risk that a larger study might detect.

The apparent discrepancy between our findings and previous reports of a more aggressive behavior of radiation-induced meningiomas may reflect the distinction between structural tumor characteristics and functional visual outcomes. Prior studies have primarily focused on multiplicity, recurrence rates, growth patterns, and histopathological features. In contrast, our study evaluated visual function. Although radiation-induced meningiomas may demonstrate more aggressive structural characteristics, they remain benign tumors whose visual effects are mediated predominantly through chronic compression of the visual pathways rather than infiltration or replacement of neural tissue. As a result, increased biological aggressiveness may not necessarily translate into worse visual outcomes. Nevertheless, because only 31 radiation-exposed patients were included in our cohort, a type II error cannot be excluded, and larger studies will be needed to determine whether subtle differences in visual prognosis exist between these groups.

It is well established that meningiomas appear at a younger age in individuals exposed to high-dose radiation [14, 22]. In our study, however, the mean age at diagnosis did not differ between patients with low-dose radiation exposure and those without. Sex distribution was similar, with over 80% women in both groups, consistent with the literature [15]. The radiation group had a higher prevalence of meningiomatosis, similar to prior reports [14]. Notably, sight-threatening skull-base meningiomas following tinea capitis irradiation were not rare, accounting for 11.4% of all such cases evaluated over the 19-year study period.

Our study has several limitations, including a small sample size in the radiation group and incomplete follow-up and imaging data for some of the patients. In particular, ganglion cell layer (GCL) measurements were available only in a small subset of patients because the study period preceded routine GCL acquisition for many examinations and because GCL analysis was not consistently available across OCT platforms. Consequently, GCL data were too sparse to permit meaningful statistical comparison. Longitudinal OCT analyses were also limited by sparse paired RNFL data. In particular, only two radiation-exposed patients with visual loss had paired RNFL measurements available, restricting our ability to draw firm conclusions regarding structural progression over time.

The radiation group had a significantly longer median delay (32 months) between meningioma diagnosis and the initial neuro-ophthalmological examination compared to controls (6.5 months). However, if anything, a longer delay would be expected to worsen visual outcomes, which was not observed in our study. Furthermore, sensitivity analyses demonstrated no significant association between the interval from meningioma diagnosis to the initial neuro-ophthalmological evaluation and either the presence of visual loss or baseline visual acuity, providing additional reassurance that this difference is unlikely to have introduced substantial bias into our findings. In addition, because the study was designed to evaluate multiple neuro-ophthalmological outcomes rather than a single predefined primary endpoint, and because the number of radiation-exposed patients was small, multivariable modeling was not performed, and residual confounding cannot be entirely excluded.

Another limitation is that the definitions of Clinical Visual Loss and Subclinical Anterior Visual Pathway Damage were investigator-defined and have not been externally validated. However, no universally accepted threshold-based definitions for compressive visual pathway injury currently exist [18–21]. To maximize specificity and minimize misclassification, the definitions used in this study incorporated conservative thresholds and required abnormalities across multiple domains of visual function and structure. Finally, the retrospective design introduces potential biases related to the completeness and consistency of clinical documentation and limits controlling for confounding factors. Despite these, this is the first study to assess visual outcomes of patients with visual pathway meningiomas after low-dose cranial irradiation.

In this cohort of patients with vision-threatening meningiomas, we did not detect statistically significant differences in clinical presentation or visual outcomes between patients exposed to low-dose childhood cranial irradiation and radiation-naïve patients. Given the relatively small number of radiation-exposed patients, particularly in the longitudinal visual-loss subgroup, modest differences between groups may have remained undetected. Importantly, because low-dose cranial or orbital irradiation continues to be used worldwide for other conditions such as thyroid eye disease [2], skin lymphomas of the head [4, 5], and others, our findings hold clinical and historical relevance beyond the specific context of Tinea capitis, These results may help guide clinicians in monitoring and counseling patients with radiation-induced meningiomas involving the visual pathways.

## Supplementary Information

Below is the link to the electronic supplementary material.

Supplementary Material 1

## Data Availability

All data supporting the findings of this study are available within the paper and its Supplementary Information.
