# Supplementary material for: Visual outcomes of sight threatening radiation-induced meningiomas after low-dose head irradiation: tinea capitis as a paradigm
Source: J Neurooncol. 2026 Jul 7;178(3):95. doi: 10.1007/s11060-026-05696-z (PMC13342210; doi:10.1007/s11060-026-05696-z)
Supplement: Supplementary file 1 — Supplementary Material 1 [file 11060_2026_5696_MOESM1_ESM.docx]

*
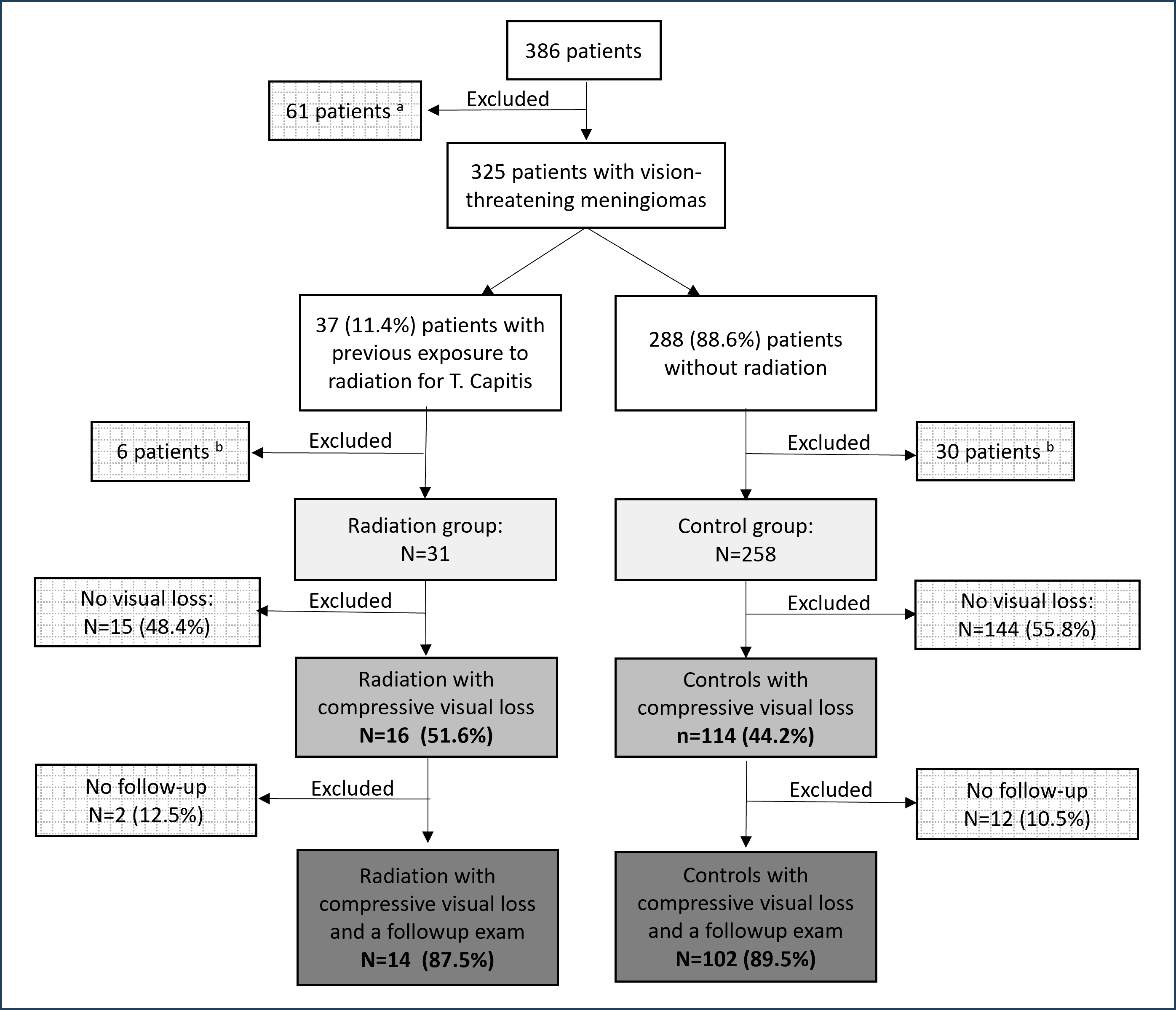
*

**Supplemental Figure 1. Study Flow Diagram Illustrating patient selection and cohort formation**

**Supplemental Fig 1 CONSORT diagram illustrating subject inclusion and exclusion processes.** After excluding 61 patients without vision-threatening meningiomas, the study cohort was categorized into two groups: those with previous radiation for T. capitis and those without. Statistical comparison between groups included only subjects with clinical or subclinical visual loss attributable to meningioma compression. For within-group comparisons of visual parameters between initial and final neuro-ophthalmological examinations, only patients with at least six months of follow-up were included. ^a^ Reasons for initial exclusion: incorrect diagnosis (n=31), meningiomas distant from visual pathway (n=20), previous brain radiation for malignant cranial tumors (n=8) and lack of documentation of previous radiation exposure (n=2). ^b^ Reasons for further exclusion: incomplete neuro-ophthalmological examination (n=1), missing data on meningioma characteristics (n=14), or visual loss due to unrelated causes confounding the diagnosis of compressive visual loss (n=21).
